# Using electroacupuncture with optimized acupoint positioning to predict the efficacy of sacral neuromodulation of refractory overactive bladder: A case report

**DOI:** 10.1097/MD.0000000000017795

**Published:** 2019-11-11

**Authors:** Jianwu Shen, Ran Luo, Lu Zhang, Yujin Li, Liupan Ke, Zhan Gao

**Affiliations:** a Department of Urology; b Department of Infertility; c Department of Acupuncture, Xiyuan Hospital, China Academy of Chinese Medical Sciences, Beijing, China.

**Keywords:** electroacupuncture, refractory overactive bladder, sacral neuromodulation, sanyinjiao acupoint, traditional Chinese medicine, zhongliao acupoint

## Abstract

**Rationale:**

Overactive bladder (OAB) is a common disease in the female urinary system. Refractory OAB is an indication for sacral neuromodulation (SNM) therapy, which was approved by the Food and Drug Administration (FDA) of the United States. However, SNM does not alleviate the clinical symptoms in all refractory OAB cases. Patients are required to undergo an SNM stage 1 operation, a traumatic and costly procedure, to evaluate the clinical efficacy of the treatment. If the procedure is predicted to likely be ineffective, the patient has to bear the physical and economic losses. Here, we report a patient with a 3-year course of refractory urge urinary incontinence who was treated with electroacupuncture according to traditional Chinese medicine (TCM).

**Patient concerns:**

The patient was 73 years old and had frequent urination and urge urinary incontinence for 3 years; she had 24 to 30 counts of urination per day and 7 to 9 counts of urge incontinence. The patient was treated with multiple TCM and Western medicines and therapies. The TCM treatment consisted of several patented Chinese medicines and TCM decoctions. The Western medication comprised mainly antibiotics, alpha receptor antagonists, and muscarinic receptor antagonists. The treatment effect was unsatisfactory, and there was no apparent alleviation of symptoms; therefore, she underwent electroacupuncture.

**Diagnosis:**

Refractory OAB.

**Interventions:**

The patient received 30 days of TCM-based electroacupuncture with optimized acupoint positioning, which comprised a total of 10 sessions (1 electroacupuncture session every 2 days) targeting the bilateral Zhongliao and Sanyinjiao acupoints. After treatment, the patient experienced a good therapeutic outcome.

**Outcomes:**

After 30 days of electroacupuncture treatment, the average daily count of urination in 5 days decreased from 29.3 per day before treatment to 19.8 after treatment, and the average count of urge incontinence decreased from 9.3 before treatment to 5.8 after treatment. However, good prognosis was not stable. After careful consideration, the patient accepted SNM treatment, which greatly alleviated the symptoms of frequent urination and urge incontinence. The patient received follow-up visits for 2 years, during which she manifested stable curative effects.

**Lessons:**

The optimized positioning at the Zhongliao acupoint improves the accuracy of acupuncture. Accurate electroacupuncture alleviates the symptoms of refractory OAB by stimulating the Zhongliao and Sanyinjiao acupoints, as the underlying mechanisms are similar to those of SNM. Therefore, it is possible to use electroacupuncture to estimate the therapeutic effect of SNM, thereby providing a reference for patients and clinicians to determine whether SNM treatment will be effective.

## 1 Introduction

Overactive bladder (OAB) is a common urological disorder. Its incidence is higher in females than in males, and it increases with age. It was reported that the incidence of OAB in adult women in suburban Beijing reached 8.1%.^[1]^ The diagnostic criteria for OAB issued by the International Continence Society were as follows: urgent urination; may be accompanied by urge urinary incontinence; frequent urination during the day (over 8 times) and night (over 1 time); and no urinary tract infection or other apparent pathological changes.^[2,3]^ Patients with refractory OAB are defined as individuals who exhibit an unsatisfactory outcome or need further therapy after 8 to 12 weeks of routine behavioral training (e.g., lifestyle guidance, bladder training, pelvic floor muscle training) and therapy with muscarinic receptor antagonists; the symptoms include frequent urination at day and night, urgent urination, and abdominal distension, which have a great impact on patients, causing tremendous pain, emotional pressure, and a feeling of inferiority.^[4]^

Anticholinergic preparation is the main conservative therapy for OAB. However, because of adverse reactions such as dry mouth, dizziness, constipation and a nonpersistent curative effect, it is of limited use. Moreover, conservative therapy does not yield satisfactory outcomes for refractory OAB and often leads to persistent and recurrent symptoms that cause extraordinary pain.^[5]^

Sacral neuromodulation (SNM) is an important treatment that was approved by the Food and Drug Administration (FDA) of the United States for treating refractory OAB. SNM uses interventional techniques to continuously apply low-frequency electrical pulses to specific sacral nerves to excite or inhibit the nerve pathways, thereby modulating abnormal sacral reflex arcs. As a consequence, the neuromodulatory approach can affect and regulate the function of target organs or regions innervated by the sacral nerve, such as the bladder and urethra pelvic floor, thereby achieving the therapeutic effect. The specific sacral nerves are mainly the third and fourth sacral nerves, which are located in the third and fourth sacral foramina.

According to classical TCM records, Baliao acupoints are the main target of acupuncture for dysuria, and Zhongliao acupoints are among the most common acupoints. In modern TCM, Baliao acupoints are located in 8 sacral foramina. Electroacupuncture stimulation of Zhongliao acupoints is highly similar to SNM. However, the present methods of modifying acupoint locations are complex. The difficulty of acupoint localization and the inadequate accuracy of acupuncture restrict the large-scale application of this technique.

In this study, we employed electroacupuncture of TCM to stimulate the Zhongliao and Sanyinjiao acupoints to treat 1 elderly female patient who had a 3-year course of neurogenic refractory OAB, which achieved good therapeutic outcomes. Our results provide a means of preoperative assessment for SNM, as the 2 treatments share similar underlying mechanisms. Afterward, the patient gladly accepted SNM and underwent a 2-year follow-up. The results revealed that the patient achieved a good lasting outcome.

## 2 Case report

The patient was a 73-year-old woman who developed symptoms of frequent urination, urgent urination, and urge urinary incontinence in 2013. She was treated in several hospitals in Beijing and was diagnosed with OAB. Accordingly, the subject orally took several drugs, including antibiotics, alpha receptor blockers, muscarinic receptor antagonists, and patented Chinese medicines, but symptom relief was not obvious. Afterward, the patient was treated in several TCM hospitals and clinics, where she was prescribed TCM decoctions. However, her symptoms showed no significant alleviation and instead were gradually aggravated. On April 17, 2016, the patient was admitted to our department. Her main complaints included a 2-year history of frequent urination (24–30 times/day), urgent urination (7–9 times/day), and urge urinary incontinence. Based on the fact that the patient had a long history of taking medicine with no obvious improvement of symptoms, she was subject to a comprehensive examination upon admission. An auxiliary examination showed negative results for the urine analysis, acid-fast staining of urine, and urine cytology. Urethra color ultrasound revealed a small bladder diverticulum, rough bladder wall, and 20 ml of residual urine. Cystoscopy revealed a slightly raised bladder neck, the formation of scattered bladder trabeculation, and a capacity of approximately 250 ml. Urodynamic examination showed the following results: the detrusor manifested unstable contraction waves; there was no urge urinary incontinence; the maximum flow rate was 15 ml/second; the detrusor pressure was 6 cm H_2_O upon the maximal flow rate; and the maximal detrusor contraction force was 14 cm H_2_O. The patient had a history of chronic gastritis. Based on these findings, the patient was diagnosed with refractory OAB. However, the patient no longer wanted to take medication; thus, we recommended that she undergo SNM. The patient was concerned about surgical pain and efficacy and refused treatment at that time.

Based on her diagnosis, the patient underwent TCM-based therapy including electroacupuncture stimulation of the Zhongliao and Sanyinjiao acupoints.

### 2.1 Treatment methods

#### 2.1.1 Treatment equipment

Hwato single-use acupuncture needles [ϕ 0.30 × 50 mm (2 inch) and ϕ 0.30 × 75 mm (3 inch)] and a Hwato SDZ-V electroacupuncture instrument were purchased from Suzhou Medical Appliance Factory (Suzhou, China).

#### 2.1.2 Needle insertion and test method

**Figure 1:**
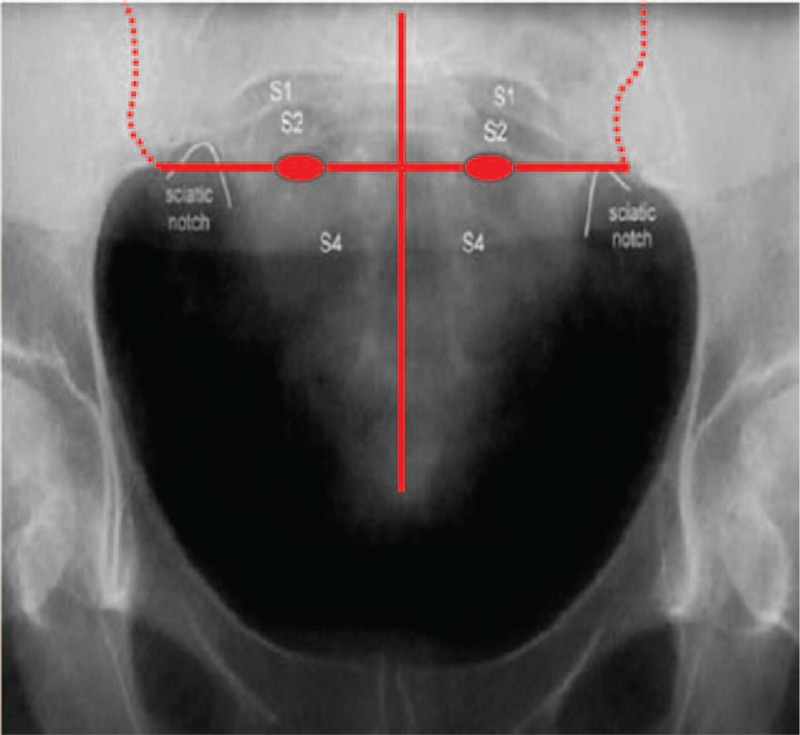
Localization of the foramen for the third sacral nerve under X-ray guidance. The vertical red line represents the posterior median line, the horizontal line represents the horizontal line of the sacral trigeminal foramen, and the 2 elliptical red dots on the horizontal line represent the specific position of the sacral trigeminal foramen. The position of the sacral trigeminal foramen was basically the same as the level of the sciatic notch.

**Figure 2:**
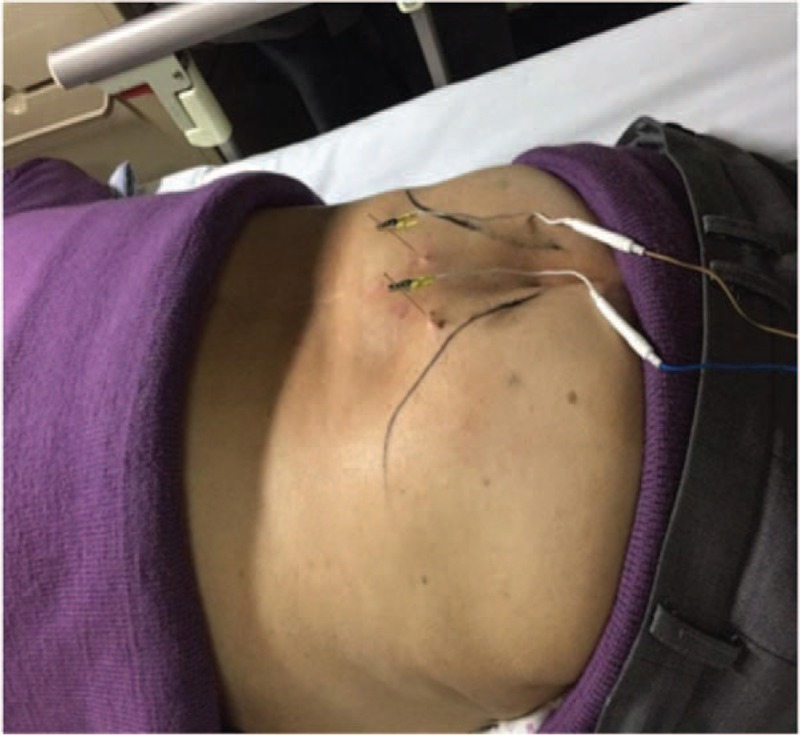
The patient after positioning and acupuncture insertion. The external part of the picture of electroacupuncture needling into bilateral sacral nerve foramen, in which the bilateral circuit is connected with the electroacupuncture therapeutic apparatus.

The patient was asked to lie in the prone position, and the local skin around the acupoints was disinfected. The optimal positioning method was adopted to locate the acupoints and was achieved by coccyx measurement as illustrated in Figures 1 and 2 (the body surface corresponding to the S3 sacral foramina was identified after measuring 9 cm upward from the tip of the coccyx along the middle line of the sacrum and 2 cm on both sides from the middle line of the sacrum). Three-inch acupuncture needles were used for the Zhongliao point. A needle was inserted 1 cm outside each S3 sacral foramen with an oblique (approximately 60°), 50-mm insertion inward and downward, before the needle was smoothly raised and twirled; the procedure was repeated 3 times. The Sanyinjiao acupoint is located on the medial side of the shank and is 3 inches above the tip of the medial malleolus and posterior to the medial margin of the tibia. A 1.5-inch needle was inserted 20 mm into each Sanyinjiao acupoint before it was raised and twirled; the procedure was repeated 3 times. At this stage, the patient felt soreness, numbness, and swelling in the area and had a sensation of Qi acquirement. Next, the electrodes of the electroacupuncture instrument were connected to the needle handles of the bilateral Zhongliao and Sanyinjiao acupoints to form an electrical circuit. The positions of the needles in the sacral foramina were adjusted by asking the patient to confirm the neurosensory responses, mainly the anal area sensation, perineal opening and closing movement (bellows-like sensation), rectal traction sensation, radiation to the vaginal labia, and plantarflexion of the ipsilateral big toe or other toes. Once the responses were detected, a continuous wave was employed that had a frequency of 30 to 40 Hz, an output current no greater than 25 mA (500 Ω load), and an output pulse width generally no greater than 0.175 ms.

The treatment cycle was as follows: 1 electroacupuncture session was provided every 3 days; 10 electroacupuncture sessions constituted a treatment cycle, which lasted for approximately 1 month; and after 1 cycle, a preliminary evaluation of the efficacy was conducted.

**Figure 3:**
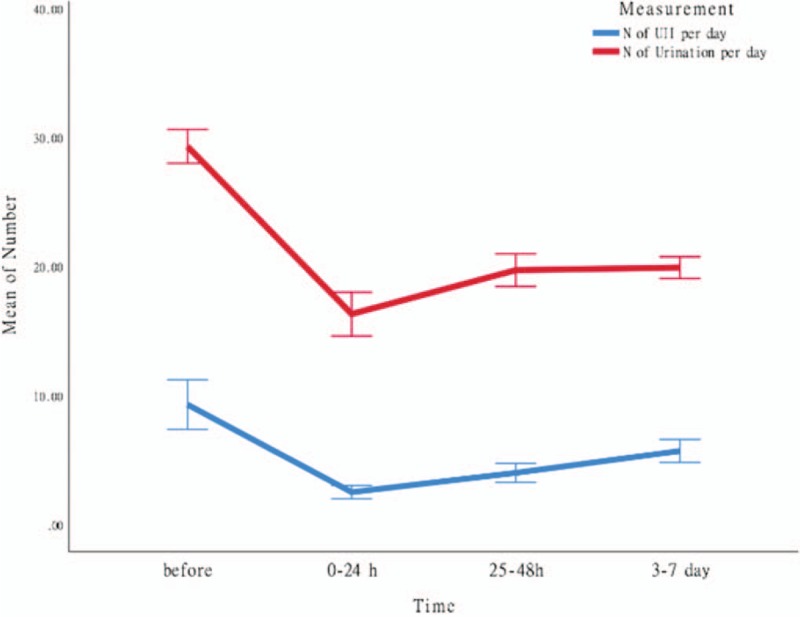
Mean symptom levels in different stages of the treatment. The red line in the picture represents the average number of times of urination per day in a given period of time; the blue line represents the average number of times of urgent urinary incontinence per day in a given period of time; the ordinate coordinate represents the number of times of urination and urinary incontinence; and the abscissa represents the different periods of observation (a total of 4 periods of observation): the first period is before treatment. Five days; the second period is 0 to 24 hours after treatment; the third period is 25 to 48 hours after treatment; the fourth period is 3 to 7 days after treatment.) From the change trend of the 2 lines, it can be seen that the frequency of urination and urinary incontinence in 0 to 24 hours after treatment is significantly lower than that before treatment. With the prolongation of treatment time, the average number of urination per day and the average number of urgent urinary incontinence per day also gradually increased. N: number. UII: urinary incontinence.

The following parameters were recorded: the average counts of urination per day and urge urinary incontinence per day in the 5 days immediately before treatment; the average counts of urination and urge urinary incontinence in the first 24 hours after each of the 10 electroacupuncture sessions; the average counts of urination and urge urinary incontinence between 25 and 48 hours after each of the 10 electroacupuncture sessions; and the average counts of urination per day and urge urinary incontinence per day from day 3 to day 7 after the entire treatment (Table 1) (Fig. 3).

**Table 1:**
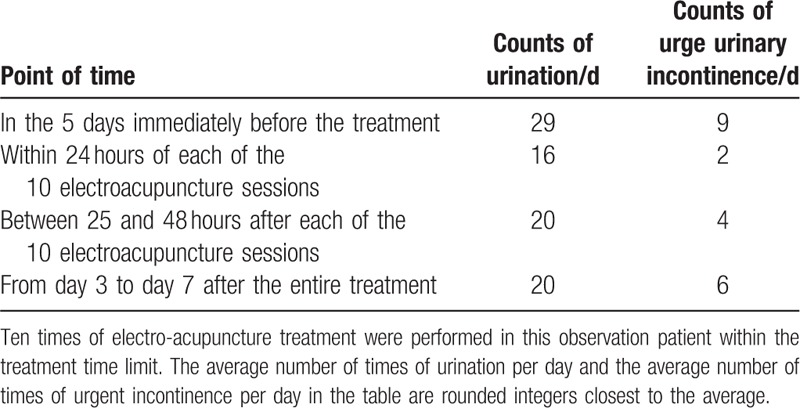
Recording at different time points about the average counts of urination per day and urge urinary incontinence per day.

No obvious discomfort symptoms and adverse reactions occurred during the treatment. After the electroacupuncture treatment, the patient showed alleviation of urination frequency and urge urinary incontinence frequency. After each session, the severity of symptoms gradually increased such that the symptoms were most improved in the first 24 hours post-session and gradually worsened from 24 to 48 hours post-session. Both the patient and physicians found that the therapeutic effect was related to the session duration as well as the duration of the entire treatment. Because the electroacupuncture could not produce lasting therapeutic effects, the patient inquired about SNM. After understanding that electroacupuncture and SNM had similar underlying therapeutic mechanisms, the patient requested SNM because she had experienced benefits from electroacupuncture. In 2016, she successfully completed the first and second stages of the SNM operation and received 2 years of follow-up visits. The results showed that the symptom relief of the patient was maintained, with the urination frequency and urge urinary incontinence frequency stabilizing at 16/day and 1 to 2/day, respectively.

## 3 Discussion

SNM generates a remission rate of 64% to 70% for the urination symptoms of refractory OAB.^[6]^ Because SNM does not always result in good therapeutic effects on refractory OAB, it is impossible for clinicians to predict whether the SNM stage 1 operation will generate a satisfactory outcome. Moreover, SNM is expensive and is typically not covered by medical insurance in China, which leads to its poor acceptance. Currently, the main therapeutic method that can predict the curative effect of SNM is tibial nerve stimulation (TNS), but the cost of TNS is high, which restricts its wide application.^[7,8]^

In TCM, refractory OAB belongs to the category of “stranguria”, “urinary incontinence” and “dysuria caused by disorder of Qi”. Acupuncture is a major TCM method used to treat this disease. Acupuncture has the advantages of being a simple operation and having a high acceptance by patients, minimal side effects, and a low cost. Baliao acupoints, especially Zhongliao, are the main targets of acupuncture for dysuria and can improve urination symptoms. The Sanyinjiao acupoint is also commonly used. Of note, the tibial nerve is located deeply behind the Sanyinjiao acupoint. As a consequence, electrical stimulation can transmit the nerve impulse to the sacral segment through the posterior tibial nerve, which produces a regulatory effect.^[9,10]^

Before receiving electroacupuncture, this patient was treated with various drugs such as Tolterodine and Solifenacin. The treatment lasted for more than half a year without obvious clinical effect. First-line drugs for overactive bladder include highly selective M-blockers such as tolterodine and Solifenacin. But about 42% of patients had no therapeutic effect on anticholinergic therapy.^[11–13]^

The second-line treatment was mainly treated with botulinum toxin type A detrusor injection and capsaicin intravesical instillation. About 40% to 50% of the patients had no obvious effect on the treatment, and the treatment needed regular repeated injection. At the same time, the incidence of patients needing intermittent cleaning catheterization after BONTA bladder injection is about 8% to 43%.^[14,15]^ This patient received the first-line drug treatment has no obvious effect, recommend the second-line treatment to this patient. Because of the fear of poor efficacy and urinary retention caused by treatment, the patient is reluctant to accept the second-line treatment. At present, SNM is the only recommended level of treatment in the third-line treatment of OAB. The effective rate of SNM in the treatment of refractory OAB is 63%.^[16]^ Because SNM is surgical treatment and expensive, when SNM was recommended to this patient, this patient was mainly concerned about poor efficacy, trauma and high cost and refuse to use it.

Therefore, we carried out the treatment of electro-acupuncture at Zhongliao and Sanyinjiao acupoints for the patient. The stimulation of the Zhongliao and Sanyinjiao acupoints by electroacupuncture can stimulate the third sacral nerve and tibial nerves, which, through the lower part of the nerve, can facilitate nerve regulation and produce therapeutic outcomes on par with those of SNM. Through electroacupuncture treatment that is simple, affordable, and well-accepted, patients can preliminarily experience a therapeutic effect similar to that of SNM, which is expensive and traumatic. Because the patients feel remarkable therapeutic effect after our characteristic electro-acupuncture treatment, the confidence of patients to receive the next surgery (SNM) treatment is enhanced, and they are willing to accept expensive SNM treatment. So after 10 times of electro-acupuncture treatment, the patient was glad to receive SNM treatment and achieved satisfactory results because he did not worry about the problem of poor curative effect. In other words, the characteristic electro-acupuncture treatment, provides a reference to determine the likelihood of success of SNM for doctors and patients.

The key to successful electroacupuncture stimulation of the Zhongliao and Sanyinjiao acupoints is accurate acupoint positioning. Sanyinjiao acupoints are easy to locate and, thus, require no elaboration. In contrast, accurate localization of the Zhongliao acupoint is complicated and is of vital importance for the therapy. Currently, localization of the Zhongliao acupoint is mostly achieved via bone landmarks, as detailed in the following. The Ciliao acupoint, located in the posterior of the pelvis, is used as the starting point. Moving from the tip of the crista iliaca inward and downward to both sides of the sacral cornua, a prominent bone process, which is the posterior superior iliac spine, can be palpated. At the middle of the sacrum is the first spinous process, which is level with the posterior superior iliac spine. Between the posterior superior iliac spine and the second spinous process of the sacrum is the second posterior sacral foramen, or the Ciliao acupoint. The Zhongliao acupoint is located in the area defined by the vertical line between the superior iliac spine and the governor meridian, which is adopted as the side from which to plot an equilateral triangle downward. The vertex of this equilateral triangle corresponds to the third posterior sacral foramen. After using this method to locate the acupoint, the patient's sensation of soreness, numbness, swelling, and Qi acquirement is used to evaluate the precision of positioning and needle insertion. However, there may be 2 problems with traditional methods. The first issue is uncertainty in acupoint positioning. Positioning based on bone landmarks is not suitable for some patients, especially obese individuals and some females who have fat hypertrophy in the buttocks. Thus, the posterior superior iliac spine cannot be completely reached by palpation. In addition, there are also some clinicians who do not have good skills in identifying bone landmarks. Both factors can lead to inaccuracy in locating the Zhongliao acupoint.

**Figure 4:**
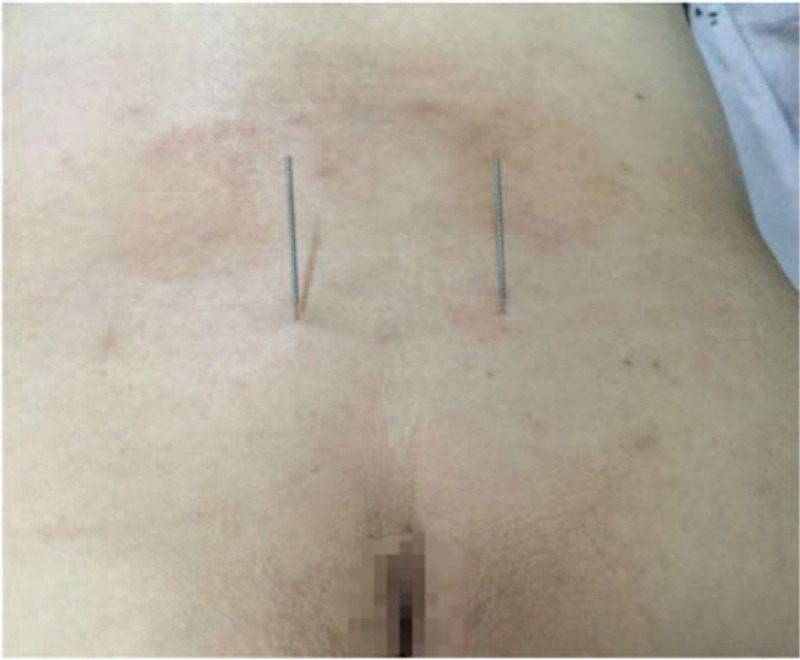
Acupuncture needles miss the bilateral sacral foramina. External part of the picture of acupuncture needles needling sacral tri-nerve foramen.

**Figure 5:**
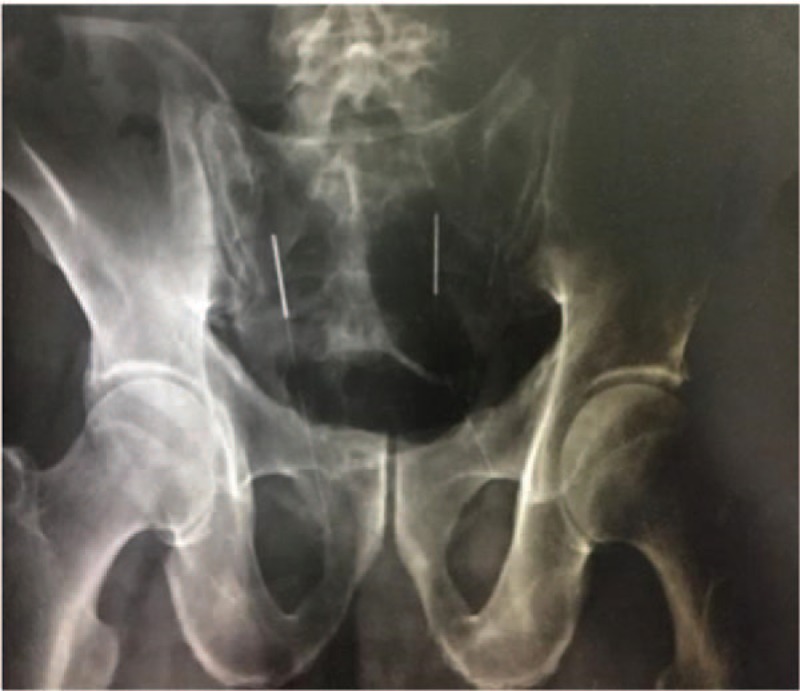
X-ray image of Fig. 4. X-ray manifestations of bilateral acupuncture needles needling bilateral sacral tri-nerve foramen were observed. Acupuncture needles on the left side of the patient accurately penetrated the sacral tri-nerve foramen, while the needles on the right side of the patient obviously bent away from the nerve foramen.

**Figure 6:**
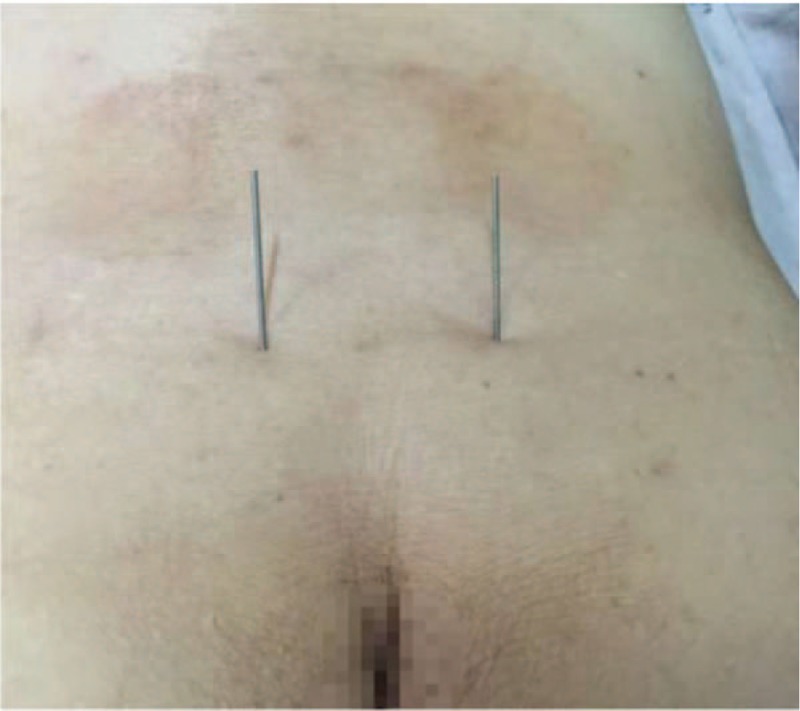
Acupuncture needles enter the bilateral sacral foramina. External part of the picture of acupuncture needles needling sacral tri-nerve foramen.

**Figure 7:**
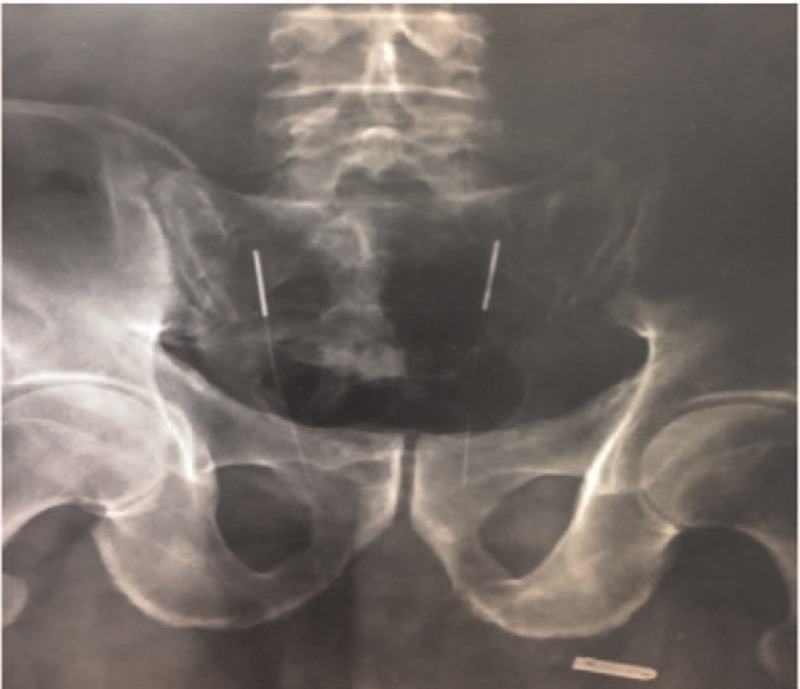
X-ray image of Fig. 6. The X-ray manifestations of bilateral sacral tri-nerve foramen were observed by bilateral acupuncture and moxibustion needles. The patients’ bilateral acupuncture and moxibustion needles penetrated the sacral tri-nerve foramen accurately.

The second issue is whether the sensation of soreness, numbness, swelling, and Qi acquirement can indeed be the indicators of precise needle insertion. The sensation of soreness, numbness, swelling, and Qi acquirement does not completely reflect successful needle entry into the sacral foramen and stimulation of the sacral nerves. Thus, such needling-related sensations are inadequate to determine the precise insertion point into the Zhongliao acupoint. Because Zhongliao is a deep acupoint and the coccygeal vertebrae have are slightly bent, a needle may not enter the sacral foramen or may slide out from the margin of the sacral foramen membrane if the needle is not long enough or if there is an angular deviation. However, the operator may still think that the needle has accurately stimulated the nerve because the patient can also experience the sensation of soreness and swelling even when the needle does not actually enter the sacral foramen. As illustrated by 1 case that our group studied previously, the appearance of needle handles on the skin surface (Figs. 4 and 6) was indistinguishable between the case where the needles missed the sacral foramina (Fig. 5) and the case where the needles entered the foramina (Fig. 7). Moreover, the patient experienced needling sensation of soreness, swelling, and numbness in both scenarios. From the X-ray image, it is obvious that the needle, which was clearly bent, did not enter the sacral foramen and slid out from the edge.

Because of the complexity and uncertainty of the traditional acupoint localization method, we herein propose an optimized positioning approach that can conveniently and accurately identify the Zhongliao acupoint. Because the location of the Zhongliao acupoint matches that of the foramen for the third sacral nerve, in this study, the TCM acupuncture localization method was integrated with the positioning method based on SNM puncturing (measuring 9 cm up from the tip of the coccyx and 2 cm from the center of the coccyx on both sides). This, in conjunction with the unique responses of different nerves, was used to accurately judge the precision of the needle entry. The motor and sensory responses to electroacupuncture stimulation of the second, third, and fourth sacral nerves were different. After needle insertion, the individual nerve foramina will produce different sensory and nerve responses. According to the different nerve responses and the simple localization method (which is easier than the TCM method), the accuracy of acupuncture at the Zhongliao acupoint (foramen for the third sacral nerve) is greatly improved, thereby ensuring that the therapeutic effect is comparable to that of SNM.

Our results revealed that the electroacupuncture stimulation of the bilateral Baliao and Sanyinjiao acupoints yielded an excellent assessment of the therapeutic effects of SNM. Of note, the TCM approach has several advantages, including no special requirement for equipment, minimal trauma, no apparent complications, and high patient acceptance. At the same time, the method allows the surgeon to conveniently choose the left side, right side, or both sides simultaneously, thereby comparing the possible effects of the stimulation options. The method provides an important basis on which to determine which side of the foramen for the third sacral nerve will generate a better therapeutic outcome once stimulated. Finally, it can potentially to be a short-term option for symptom improvement and pain alleviation in patients who cannot afford SNM therapy.

## Author contributions

**Conceptualization:** Jianwu Shen, Ran Luo.

**Data curation:** Lu Zhang.

**Funding acquisition:** Jianwu Shen.

**Methodology:** Ran Luo.

**Resources:** Zhan Gao.

**Software:** Yujin Li, Liupan Ke.

**Supervision:** Ran Luo.

**Validation:** Jianwu Shen.

**Visualization:** Jianwu Shen.

**Writing – original draft:** Jianwu Shen.

**Writing – review & editing:** Jianwu Shen.
